# Isolation of Plasma Membrane Vesicles from Mouse Placenta at Term and Measurement of System A and System β Amino Acid Transporter Activity

**DOI:** 10.1016/j.placenta.2009.11.006

**Published:** 2010-01

**Authors:** L.C. Kusinski, C.J.P. Jones, P.N. Baker, C.P. Sibley, J.D. Glazier

**Affiliations:** Maternal and Fetal Health Research Group, School of Clinical and Laboratory Sciences, University of Manchester, St. Mary's Hospital, Oxford Road, Manchester M13 9WL, United Kingdom

**Keywords:** Amino acid, Placental transport, Mouse, Trophoblast, Taurine, Alkaline phosphatase

## Abstract

Placental amino acid transport is essential for optimal fetal growth and development, with a reduced fetal provision of amino acids being implicated as a potential cause of fetal growth restriction (FGR). Understanding placental insufficiency related FGR has been aided by the development of mouse models that have features of the human disease. However, to take maximal advantage of these, methods are required to study placental function in the mouse. Here, we report a method to isolate plasma membrane vesicles from mouse placenta near-term and have used these to investigate two amino acid transporters, systems A and β, the activities of which are reduced in human placental microvillous plasma membrane (MVM) vesicles from FGR pregnancies. Plasma membrane vesicles were isolated at embryonic day 18 by a protocol involving homogenisation, MgCl_2_ precipitation and centrifugation. Vesicles were enriched 11.3 ± 0.5-fold in alkaline phosphatase activity as compared to initial homogenate, with minimal intracellular organelle contamination as judged by marker analyses. Cytochemistry revealed alkaline phosphatase was localised between trophoblast layers I and II, with intense reaction product deposited on the maternal-facing plasma membrane of layer II, suggesting that vesicles were derived from this trophoblast membrane. System A and system β activity in mouse placental vesicles, measured as Na^+^-dependent uptake of ^14^C-methylaminoisobutyric acid (MeAIB) and ^3^H-taurine respectively confirmed localisation of these transporters to the maternal-facing plasma membrane of layer II. Comparison to human placental MVM showed that system A activity was comparable at initial rate between species whilst system β activity was significantly lower in mouse. This mirrored the lower expression of TAUT observed in mouse placental vesicles. We conclude that syncytiotrophoblast layer II-derived plasma membrane vesicles can be isolated and used to examine transporter function.

## 1 Introduction

Adequate provision of amino acids is required for optimal fetal development [1]. Altered amino acid supply to the fetus may significantly impact on development and lead to fetal growth restriction (FGR), a condition associated with high levels of perinatal mortality and morbidity [2] including neurodevelopmental delay and handicap in those surviving [3]. FGR is a condition associated with various aetiologies [4]. However, over recent years there has been growing evidence to suggest that impaired nutrient transfer across the placenta directly limits fetal growth [4,5]. In FGR there is a reduction in cord plasma concentration of amino acids including essential amino acids [6,7], and this is associated with a reduced activity of a range of amino acid transport systems in both the microvillous (MVM) and basal (BM) plasma membranes of the syncytiotrophoblast [5]. These include system A and system β transporters in MVM [8–11], system L transporter in MVM and BM [12] and system y^+^L transporter in BM [12].

In human placental MVM, the magnitude of the reduction in system A activity relates to the severity of FGR [8], but definitive evidence that impaired system A activity is causal in inducing FGR has come from animal models. Cramer et al. [13] used *N*-methylated amino acid α-(methylamino)isobutyric acid (MeAIB), a non-metabolisable substrate for system A [14], to inhibit placental system A activity in rats thereby impacting on placental delivery of endogenous neutral amino acid substrates to fetuses. This manipulation resulted in a reduced maternofetal transfer of radiolabelled MeAIB, reduced system A activity in isolated apical plasma membrane vesicles and a significant reduction in fetal weight near-term. Other studies, using dietary manipulation in pregnant rats to induce FGR, have clearly demonstrated that the downregulation in placental system A activity precedes the onset of FGR [15]. These observations clearly delineate system A as a key transporter in determining fetal growth. Likewise, the importance of system β amino acid transporter, encoded by the TAUT gene, in promoting normal growth is exemplified by the observation that mice homozygous for the deletion of the TAUT gene (*taut*^−/−^) are significantly smaller than their wild-type siblings, and do not exhibit catch-up growth [16].

These studies emphasise the importance of using rodent models, which can be relatively easily manipulated, to elucidate the role of specific placental amino acid transporters in the pathogenesis of FGR. Additionally, mouse genetic variants that represent conditions prevalent in humans are important in advancing our understanding of disease processes. As a prelude to examining placental amino acid transporter activity in genetically modified mice, the aim of this study was to isolate plasma membrane vesicles from a defined trophoblast plasma membrane of the near-term mouse haemotrichorial placenta and to assess whether these could be used to investigate the activities of system A and system β amino acid transporters as key determinants of fetal growth and development. To do this we adapted the Mg^2+^-chelation method used to isolate plasma membrane vesicles from rat placenta [17], based on the original method to prepare MVM vesicles from human placenta [18]. In order to establish the suitability of alkaline phosphatase as a marker of purity for the mouse placental vesicle preparation, cytochemical localisation of this enzyme in mouse placenta was performed. Finally, we compared the activities of system A and system β amino acid transporters in mouse placental vesicles with that in human placental MVM vesicles.

## 2 Methods

### 2.1 Chemicals

All chemicals were purchased from either Sigma–Aldrich Co. Ltd (Poole, UK) or VWR International (Lutterworth, UK) unless otherwise stated.

### 2.2 Animals

C57/B1 mice were mated and the first day of gestation determined by the discovery of a copulation plug (term is 19–20 days). All animals were provided with nesting material and housed in cages maintained under constant 12 h light–dark cycle at 21–23 °C with free access to food (Beekay Rat and Mouse Diet, Bantin & Kingham, Hull, UK) and tap water. All animals were killed by Schedule 1 procedure in accordance with the UK Animals (Scientific Procedures) Act of 1986.

### 2.3 Alkaline phosphatase localisation in mouse placenta

Alkaline phosphatase localisation was performed as described previously [17]. Briefly, placentas were collected from an individual mouse at embryonic day 18 (E18) and cut into four sections. Tissue was fixed by immersion in 2.5% glutaraldehyde in 0.1 M sodium cacodylate buffer (pH 7.4) for 4 h followed by washing in cacodylate buffer containing 3 mM calcium chloride. Thin slivers of tissue were cut with a razor blade, cut into small squares and washed in 0.2 M Tris–HCl buffer (pH 8.5) followed by incubation in the presence or absence (negative control) of substrate as described previously [19]. After rinsing thoroughly with Tris–HCl buffer (pH 8.5), tissue was washed with distilled water and cacodylate buffer, post-fixed with 1% osmium tetroxide for 1 h, dehydrated in a graded alcohol series and propylene oxide and embedded in TAAB epoxy resin (TAAB Laboratories Equipment Ltd, Aldermaston, UK). Ultrathin sections were cut and mounted on 300 mesh copper grid and examined at an accelerating voltage of 80 kV in a Phillips CM10 electron microscope without counterstaining. Digital images were captured using a Deben camera and stored as 4 MB TIFF files.

### 2.4 Vesicle preparation and purification

For isolation of mouse placental vesicles, a modification of the method to prepare rat placental vesicles was followed [17]. Placentas were collected from 2–6 litters at E18 and placed on ice. Tissue was weighed (1.05–3.93 g) and homogenised for approximately 30 s in 5 volumes (w/v) ice-cold mannitol buffer (300 mM mannitol, 10 mM Hepes–Tris, 1 mM MgSO_4_, pH 7.4). A sample (30 μl) of the homogenate was retained for enzyme activity and protein analysis, and to the remaining homogenate 12 mM MgCl_2_ was added; this was stirred on ice for 10 min. The homogenate was then centrifuged at 2300×g for 15 min and the supernatant retained and centrifuged at 23,500×g for 40 min. The resulting pellet was resuspended in intravesicular buffer (290 mM sucrose, 5 mM Tris, 5 mM Hepes, pH 7.4) using a Dounce homogenizer and the vesicle suspension was passed 15–20 times through a 25G syringe needle to yield the vesicle suspension. For comparison, human placental MVM vesicles were prepared by a protocol employing two rounds of Mg^2+^-precipitation as described previously [18]. Vesicles intended for Western blot analysis were stored at −80 °C.

Protein content of mouse homogenate and vesicle fractions was measured by the method of Bradford [20] using a commercial kit (Biorad, Hertfordshire, UK). Alkaline phosphatase activity was measured at pH 9.8 using p-nitrophenyl phosphate as substrate [21]. Vesicle purity was further assessed by measuring NADH dehydrogenase [22] and succinate dehydrogenase [23] activities as markers of endoplasmic reticulum and mitochondrial contamination respectively, as previously described [17].

### 2.5 System A and system β activity in mouse placental vesicles

All measurements of system A and system β activity were performed within one day of mouse vesicle isolation. System A activity was measured over 1 min at room temperature (∼22 °C) as previously described in the presence and absence of an inwardly directed Na^+^ gradient [10]. The Na^+^-dependent component of ^14^C-MeAIB uptake was taken to indicate System A activity. Briefly, uptake was initiated by the addition of 20 μl mouse vesicles (124–281 μg protein) to 20 μl ^14^C-MeAIB (0.33 mM; Perkin Elmer, Buckinghamshire, UK) in the presence or absence of Na^+^ contained in the extravesicular buffer (5 mM Tris, 5 mM Hepes, 145 mM NaCl or KCl, pH 7.4) [10]. Vesicular uptake was stopped by the addition of 2 ml ice-cold Krebs Ringer phosphate buffer (KRP; 130 mM NaCl, 10 mM Na_2_HPO_4_, 4.2 mM KCl, 1.2 mM MgS0_4_, 0.75 mM CaCl_2_, pH 7.4) and 2 ml of the resultant solution applied to a filter under vacuum filtration, which was washed with 10 ml KRP and dissolved in 2 ml 2-ethoxyethanol and counted by liquid scintillation spectroscopy. System β activity was determined in a similar manner using 20 μl ^3^H-taurine (1 μM; GE Healthcare, Buckinghamshire, UK) as substrate. Non-specific binding of tracer to vesicle plasma membrane was quantified by incubation of vesicles with 0.2% Triton to disrupt vesicle integrity. Specificity of transporter activities was also examined by competitive inhibition using model substrates for each transport mechanism (20 mM for system A and 500 μM for system β) in the uptake buffer. For comparison, uptakes were also performed using human placenta MVM vesicles, isolated using magnesium precipitation as described previously [18] and of high purity as judged by alkaline phosphatase enrichment (21.9 ± 1.6-fold; *n* = 8).

### 2.6 Western blot analysis of TAUT expression

Human MVM and mouse vesicle samples (40 μg protein) were mixed with sample buffer (8 M urea, 5% SDS, 455 mM dithiothreitol, 0.04% bromophenol blue in 50 mM Tris–HCl, pH 6.9) in a 2:1 volume ratio (vesicles:sample buffer). Mouse kidney lysate (Santa Cruz Biotechnology, California, USA) was included as positive control [24]. The samples were subjected to SDS-PAGE on a 8% gel and were then electrotransferred to nitrocellulose membranes. The membranes were probed with an affinity-purified rabbit polyclonal antibody raised against rat TAUT (1:250; Alpha Diagnostic International, San Antonio, Texas) for 2 h at room temperature, using similar conditions to that described previously [25]. To confirm antibody specificity, parallel negative controls were included whereby incubations were performed with primary antibody that had been pre-adsorbed with 10× antigenic peptide overnight at 4 °C. Blots were re-probed for β-actin (1:1000; Abcam, Cambridge, England) to confirm comparable protein loading and preservation of sample integrity.

### 2.7 Statistics

Data is presented as the mean ± SEM, with *n* = number of preparations (each preparation being pooled from ≥2 litters). Statistical analysis using least-squares linear regression analysis, one-way ANOVA followed by a Dunnett's multiple comparison test or two-way ANOVA followed by a Bonferroni post-test were applied as appropriate with *p* < 0.05 taken to be significant.

## 3 Results

### 3.1 Alkaline phosphatase ultracytochemistry

**Fig. 1:**
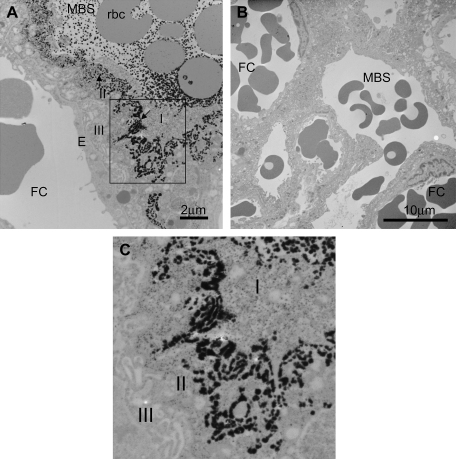
Alkaline phosphatase distribution in mouse placenta. (A) Intense reaction product, indicative of alkaline phosphatase activity, was localised between trophoblast layers I and II (black arrows) with deposits clearly visible between the plasma membrane infoldings of layer II. The boxed area is magnified in Fig. 1C. (B) No reaction product was observed in the absence of substrate. (C) Reaction product distribution within layer II. I, II, III indicate the three trophoblast layers; FC, fetal capillary; E, fetal capillary endothelium; MBS, maternal blood space; rbc, red blood cell.

Intense deposits of reaction product indicative of alkaline phosphatase activity were predominantly localised between syncytial layers I and II, with no reaction product visible in trophoblast layer III or fetal capillary endothelium (Fig. 1A and C). Reaction product was clearly associated with plasma membrane infoldings of layer II (Fig. 1C) and was also associated with plasma proteins and maternal erythrocytes within the maternal blood space (Fig. 1A). No reaction product was observed in the absence of substrate (Fig. 1B).

### 3.2 Protein recovery and purity in mouse placental vesicles

The amount of protein recovered in the mouse placental membrane vesicle fraction from pooled placentas of ≥2 litters was 3.5 ± 0.3 mg/g placenta (*n* = 22). Marker enzyme activities in homogenate and vesicle fractions are shown in Table 1. Alkaline phosphatase activity was markedly enriched in mouse placental vesicles. In contrast, there was no enrichment of NADH dehydrogenase or succinate dehydrogenase activities indicating minimal contamination with endoplasmic reticulum and mitochondria respectively.

### 3.3 System A uptake in mouse placental vesicles

**Fig. 2:**
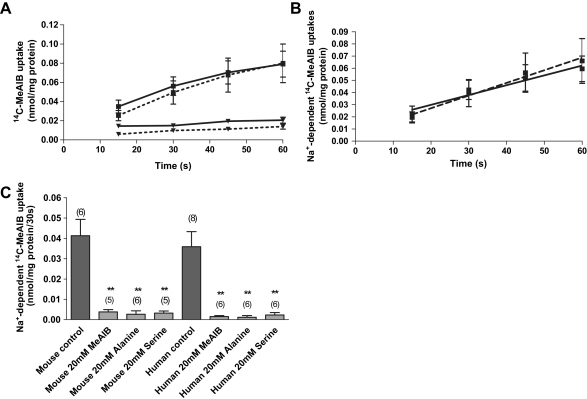
Uptake of ^14^C-MeAIB (0.165 mM) into mouse placental vesicles. (A) Uptakes were measured in the presence (■) and absence (▾; K^+^ replacement) of an inwardly directed Na^+^ gradient over 60 s. (B) Linearity of Na^+^-dependent ^14^C-MeAIB uptake into mouse placental (*r*^2^ = 0.95, *p* < 0.05, solid line; *n* = 6) and human MVM vesicles (*r*^2^ = 0.98, *p* < 0.05, dashed line; *n* = 6) over 60 s. The gradients of the lines were not significantly different (*F* test). Data are expressed as mean ± SEM. (C) Effect of neutral amino acids (20 mM) on Na^+^-dependent ^14^C-MeAIB uptake into mouse placental and human MVM vesicles. Data is expressed as mean + SEM with (*n*) given above bars. In both mouse and human vesicles uptake of ^14^C-MeAIB was significantly reduced by each amino acid (*p* < 0.01, One-way ANOVA with Dunnett's multiple comparison test).

Uptake of ^14^C-MeAIB into mouse placental vesicles was time-dependent with uptake in the presence of Na^+^ significantly higher (*p* < 0.0005, 2-way ANOVA with Bonferroni's post-test) than that in the absence (with K^+^ replacement) at all time points (Fig. 2A). Na^+^-dependent uptake of ^14^C-MeAIB in mouse vesicles was linear over 60 s (Fig. 2B). Uptake of ^14^C-MeAIB reflected accumulation within an intravesicular space as uptake was negligible in the presence of 0.2% Triton (data not shown). The gradient of the linear regression line was not significantly different between mouse placental and human MVM vesicles measured under the same conditions (Fig. 2B). Fig. 2C shows the competitive inhibition of ^14^C-MeAIB by an excess of the unlabelled neutral amino acids, representing model substrates of system A, measured at 30 s (taken to be initial rate). In the presence of each amino acid, uptake into mouse placental vesicles was significantly reduced, with a similar pattern of inhibition observed in human MVM vesicles (Fig. 2C).

### 3.4 System β uptake in mouse placental vesicles

**Fig. 3:**
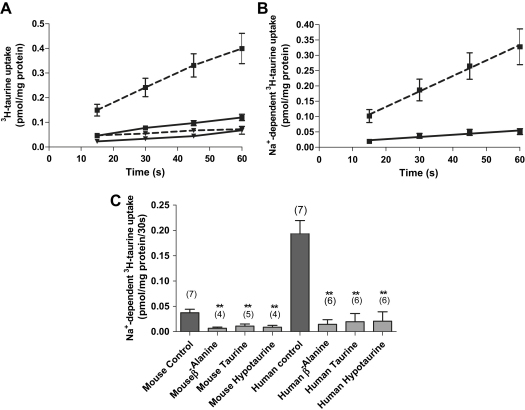
Uptake of ^3^H-taurine (0.5 μM) into mouse placental vesicles. (A) Uptakes were measured in the presence (■) and absence (▾; K^+^ replacement) of an inwardly directed Na^+^ gradient over 60 s. (B) Linearity of Na^+^-dependent ^3^H-taurine uptake into mouse placental (*r*^2^ = 0.90, *p* < 0.05, solid line; *n* = 6) and human MVM vesicles (*r*^2^ = 1.0, *p* < 0.005, dashed line; *n* = 5) over 60 s. The gradients of the lines were significantly different (*p* < 0.0005, *F* test). Data are expressed as mean ± SEM. (C) Effect of β-amino acids (500 μM) on Na^+^-dependent ^3^H-taurine uptake into mouse placental and human MVM vesicles. Data is expressed as mean + SEM with (*n*) given above bars. In both mouse and human vesicles uptake of ^3^H-taurine was significantly reduced by each amino acid (*p* < 0.01 One-way ANOVA with Dunnett's multiple comparisons test).

Uptake of ^3^H-taurine into mouse placental vesicles increased with time over 15–60 s, with uptake in the presence of Na^+^ significantly higher (*p* < 0.001, 2-way ANOVA with Bonferroni's post-test) than that in the absence of Na^+^ at all time points (Fig. 3A). Na^+^-dependent uptake of ^3^H-taurine in mouse vesicles was linear over 60 s (Fig. 3B) and into an intravesicular space, as in the presence of 0.2% Triton, uptake was negligible (data not shown). In contrast to system A, both the magnitude and rate of ^3^H-taurine uptake into mouse placental vesicles over 60 s was markedly lower than in human placental MVM vesicles (Fig. 3B). Fig. 3C shows the competitive inhibition of ^3^H-taurine by excess unlabelled β-amino acids, as model substrates of system β measured at initial rate (30 s). In the presence of each amino acid, uptake into mouse placental vesicles was significantly reduced, similar to the pattern of inhibition observed in human placental MVM vesicles (Fig. 3C).

### 3.5 TAUT protein expression in mouse placental vesicles

**Fig. 4:**
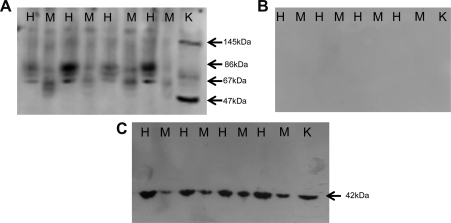
Western blot comparing TAUT expression in mouse placental vesicles to human placental MVM. (A) A representative Western blot of four mouse placental vesicle isolates (M) and MVM isolates from human placenta (H) probed for TAUT, with mouse kidney lysate (K) included as positive control. Protein loading was 40 μg/lane other than mouse kidney lysate (25 μg). An immunoreactive signal was seen in all human MVM lanes with a molecular weight of ∼86 and ∼67 kDa. These bands were less intense in mouse placental vesicles. Signals were also observed in mouse kidney lysate at ∼145 and ∼47 kDa. Film exposure was 30 min. (B) Negative control showing that all TAUT signals were abolished by excess blocking peptide. (C) The same blot re-probed for β-actin showing immunoreactive signal in all samples. Film exposure was 5 min.

To investigate the lower system β activity in mouse placental vesicles further, the expression of the taurine transporter (TAUT) which mediates this activity was examined (Fig. 4). Fig. 4A shows a representative Western blot that compares the expression of in mouse placental vesicles to that in human placental MVM. Confirmation that the primary antibody was immunoreactive with murine TAUT protein was evidenced by the signal detected in mouse kidney as positive control (Fig. 4A). Multiple TAUT species were observed in both human MVM and mouse placental vesicles, with signal intensity relatively more pronounced in human placental MVM (Fig. 4A). All immunoreactive signals were abolished by pre-adsorption of the antibody with 10× excess antigenic peptide (Fig. 4B), confirming antibody specificity. Reprobing of the blot for β-actin (Fig. 4C), confirmed protein integrity in all samples.

## 4 Discussion

Our observation that alkaline phosphatase activity in the near-term mouse placenta is predominantly localised to the maternal-facing plasma membrane of syncytiotrophoblast II layer is consistent with previous reports in this species describing the presence strong alkaline phosphatase activity to numerous infoldings in layer II with minimal activity localised to either the apical or basal plasma membrane of layer I [26]. This predominant distribution of alkaline phosphatase to the maternal-facing plasma membrane of syncytiotrophoblast layer II in mouse placenta mirrors that found in rat placenta [17], suggesting a common pattern of polarised alkaline phosphatase activity between these two species with haemotrichorial placentation.

This polarisation of alkaline phosphatase activity is also reflected in the haemomonochorial human placenta with the predominant localisation of alkaline phosphatase to the MVM plasma membrane of the syncytiotrophoblast [19]. These data therefore raise the possibility that the maternal-facing plasma membrane of syncytiotrophoblast layer II in rodent placenta and the MVM plasma membrane in human syncytiotrophoblast possess common catalytic properties and complement of intrinsic proteins. In support of this concept, maternal-facing plasma membranes from trophoblast layer II of rat placenta and human placental MVM have several transport proteins found in common; Na^+^/H^+^ exchanger [17], lactate transporter [27], and systems A [28–30], ASC [31], ${\text{X}}_{\text{AG}}^{-}$ [31,32], y^+^ and y^+^L [31] amino acid transporters.

Collectively, these observations strongly suggest that alkaline phosphatase activity is predominantly associated with, and a relatively good marker of, the first trophoblast plasma membrane to confer restriction to maternofetal transfer of solutes. This proposal is supported by permeability studies which have demonstrated that tracers such as horseradish peroxidase penetrated trophoblast layer I relatively rapidly in both rat [33] and mouse [26] placenta, collecting in the space between layers I and II, whereas further movement into trophoblast layer II was minimal. This supports the concept that the maternal-facing plasma membrane of trophoblast layer II represents the first major plasma membrane barrier to maternofetal transport.

The usefulness of the Mg^2+^-precipitation and differential centrifugation approach employed here to isolate apical plasma membranes from placenta relies on there being a contiguous anionic charge within the plasma membrane, which allows cross-linkage with Mg^2+^ without aggregation, in contrast to other non-apical plasma membranes [34]. The effectiveness of this approach in isolating MVM in a relatively pure form, as judged by marker analysis, from human syncytiotrophoblast [18], the maternal-facing plasma membrane from trophoblast layer II of rat [17,28,30,35] and mouse placenta as shown here, implies that all these trophoblast plasma membranes have a highly negative surface charge density in common.

In this study we elected to use one round of Mg^2+^-precipitation, rather than the two rounds previously employed for isolating human MVM [18] and rat [17] placental membrane vesicles. The reason for this was concern that the amount of protein recovered after a second round of Mg^2+^-precipitation and differential centrifugation steps would be inadequate for performing subsequent marker and transport assays; only about 8% of the protein present after one Mg^2+^-precipitation treatment is recovered following a second Mg^2+^-precipitation and differential centrifugation cycle [17]. However, in the isolation of rat placental membrane vesicles [17] it is the second Mg^2+^-precipitation cycle (at 10 mM) that improves the purity of the preparation [17]. To mitigate against this in the current study, the concentration of Mg^2+^ in the single cycle treatment was increased to 12 mM. This protocol appeared to be effective as judged by the enrichment of alkaline phosphatase as a purity marker, which accords well with rat placental membrane preparations (10.6–13.6-fold; [17,30]) and the observation of others employing a 12 mM MgCl_2_ purification step to isolate mouse placental membranes (13-fold; [36]). Furthermore, the single magnesium purification step was effective in limiting contamination from plasma membranes of intracellular organelles as evidenced by the lack of enrichment in markers of the endoplasmic reticulum and mitochondria.

Our data support preservation of functional activity within the isolated mouse placental membrane vesicle fraction. We were able to demonstrate the presence of Na^+^-dependent system A amino acid transporter activity using radiolabelled MeAIB as substrate. Importantly, our data show that this activity was similar to human placental MVM vesicles at initial rate and could be competitively inhibited by model system A substrates. System A activity is mediated by three highly homologous isoforms of the sodium-coupled neutral amino acid transporter (SNAT) family, namely SNAT1, SNAT2 and SNAT4 which are encoded by the genes SLC38A1, SLC38A2 and SLC38A4 respectively [37]. All three SNAT isoforms, SNAT1, SNAT2 and SNAT4, are expressed in mouse placenta, at least at the mRNA level [38], in common with rat [15,39] and human [40] placenta. SNAT1 and SNAT2 protein show prominent distribution to rat labyrinth [39] whilst SNAT1 [41], SNAT2 [25,41] and SNAT4 [40,41] are localised to human syncytiotrophoblast MVM. It is unclear which of these three isoforms contribute to the activity demonstrated here in mouse placental vesicles but the recent localisation of SNAT2 and SNAT4 to the maternal-facing plasma membrane of syncytiotrophoblast layer II of mouse placenta makes these attractive candidates [36].

Taurine transport across human syncytiotrophoblast plasma membranes is mediated by system β amino acid transporter [11], a Na^+^- and Cl^−^ dependent transporter which also accepts other β-amino acids such as β-alanine and hypotaurine as substrates [42–44]. Based on these characteristics, we have confirmed the presence of system β transporter in mouse placental vesicles derived from the maternal-facing plasma membrane of syncytiotrophoblast layer II. Uptake of taurine into mouse placental vesicles at initial rate was Na^+^-dependent but of significantly lower magnitude compared to human placental MVM. This cannot be accounted for by either a lower purity or a greater dissipation of the Na^+^ gradient in mouse placental vesicles compared to human placental MVM as this effect was not observed for system A uptakes performed in parallel. We and others, have previously demonstrated that TAUT protein is localised to the MVM of human syncytiotrophoblast [25,45,46]. Here we demonstrate that TAUT is also distributed to the maternal-facing plasma membrane of syncytiotrophoblast layer II. Interestingly, several TAUT species were detected in mouse placental vesicles, similar, in part, to the banding pattern observed in human placental MVM. This may reflect the existence of differentially glycosylated TAUT variants in the plasma membrane of syncytiotrophoblast layer II [45]. The trend towards a less intense TAUT signal in mouse placental vesicles compared to human placental MVM would accord with the lower system β activity measured in mouse vesicles. There is also the possibility is that the kinetic properties of system β are different between the maternal-facing plasma membrane of syncytiotrophoblast layer II of mouse placenta and human syncytiotrophoblast MVM. This suggestion is consistent with the observation that the affinity (*K*_m_) for this transport mechanism varies by more than two orders of magnitude between different tissues and species [47].

The similarity between system A uptake in mouse placental vesicles compared to human placental MVM vesicles at initial rate, suggests the mouse might be a useful model to further investigate the activity of placental system A, particularly as its activity is downregulated in both human placental MVM [8–10] and rodent placenta [15,38] in pregnancies compromised by FGR. In contrast, the lack of correspondence between the activity of system β in mouse placental vesicles and human placental MVM limits this species' utility in examining the mechanisms underlying the reduced system β activity associated with FGR [11].

In summary, we have established a protocol for isolating the maternal-facing plasma membrane of trophoblast layer II of mouse placenta, as judged by the enrichment of alkaline phosphatase in this membrane fraction and the lack of contamination from other plasma membranes of intracellular organelles. We have demonstrated comparability in system A activity at initial rate between mouse syncytiotrophoblast layer II vesicles and human syncytiotrophoblast MVM vesicles, contrasting with the disparate system β activity observed in plasma membranes isolated from these species. We have also demonstrated that TAUT protein expression in mouse placental vesicles is relatively low as compared to human placental MVM, consistent with the lower system β activity observed in the former. This study suggests that isolated mouse placental plasma membrane vesicles may be useful to examine system A-induced FGR, and further, placental transport dysfunction in genetically modified mice.

## Figures and Tables

**Table 1:** Marker enzyme activities in mouse placental homogenate and vesicles.

	Homogenate	Vesicles
Alkaline phosphatase (μmol product/mg protein/min)	0.96 ± 0.15 (22)	8.94 ± 0.83 (22)
Alkaline phosphatase enrichment		11.3 ± 0.5
NADH dehydrogenase (μmol product/mg protein/min)	2.15 ± 0.55 (3)	2.05 ± 0.54 (3)
NADH dehydrogenase enrichment		0.95 ± 0.02
Succinate dehydrogenase (nmol product/mg protein/min)	14.3 ± 10.4 (3)	7.8 ± 7.2 (3)
Succinate dehydrogenase enrichment		0.16 ± 0.2

Mean ± SEM (*n*) is shown.
